# Prevalence and characteristics of scoliosis among ethiopian schoolchildren aged 6–15 Years: A school-based cross-sectional study

**DOI:** 10.1371/journal.pone.0344671

**Published:** 2026-03-20

**Authors:** Reta Wakoya, Mekbeb Afework, Alemayehu Worku, Firaol Dandena, Stefano Bolongaro, Timothy Nunn

**Affiliations:** 1 Department of Biomedical Science, Menelik II Medical and Health Science College, Addis Ababa, Ethiopia; 2 Department of Anatomy, School of Biomedical and Laboratory Medicine, College of Health Science, Addis Ababa University, Addis Ababa, Ethiopia; 3 School of Public Health, Addis Ababa University, Addis Ababa, Ethiopia; 4 CURE Children’s Hospital of Ethiopia, Addis Ababa, Ethiopia; Lorestan University, IRAN, ISLAMIC REPUBLIC OF

## Abstract

**Background:**

Scoliosis is a progressive spinal deformity that often develops during childhood and adolescence. In Ethiopia, population-level prevalence data are scarce, and school-based screening, though practical, may overestimate cases without radiographic confirmation. Understanding its distribution and severity is critical for guiding clinical and public health strategies.

**Objectives:**

To estimate the prevalence of scoliosis among Ethiopian schoolchildren, characterize its types and severity, and examine associations with clinical and anthropometric variables.

**Methods:**

A cross-sectional school-based screening was conducted from March 2024 to June 2025 across 42 public primary schools in six regions. Children aged 6–15 years were screened using the Adam’s Forward Bend Test and scoliometer; suspected cases (ATR ≥ 7°) were referred for radiographic confirmation. Prevalence estimates and associations were analyzed using chi-square tests and t-tests. Data were analyzed in Python, with quality control ensured through standardized training, pilot testing, and double-entry verification.

**Results:**

Of 32,000 children screened, 48 were suspected of scoliosis (0.15%; 95% CI: 0.11–0.20%), and 21 were radiographically confirmed (0.066%; 95% CI: 0.04–0.10%). Congenital scoliosis was most common (61.9%), with male predominance (69%), while idiopathic scoliosis (23.8%) was more frequent in females (60%). Neuromuscular and syndromic scoliosis were rare. Severity analysis showed male predominance in mild and very severe cases, with equal sex distribution in severe scoliosis. The mean Cobb angle was 47.4° (SD ± 28.9), most cases involved the thoracic spine (52.4%), and the rib hump was typically right-sided (61.9%). Cobb angle correlated positively with ATR (r = 0.61) and thoracic loss (r = 0.48), and negatively with age (r = –0.24) and thoracic height (r = –0.46).

**Conclusion:**

This study shows that scoliosis prevalence within the school population is low, with adolescent idiopathic cases markedly underrepresented compared to other school-based screening reports. These findings suggest that nationwide school screening programs are not recommended. Instead, efforts should prioritize strengthening diagnostic and referral pathways for clinically evident cases to ensure timely access to specialized care.

## 1. Background

Scoliosis is a structural spinal deformity characterized by a lateral curvature exceeding 10°, typically accompanied by vertebral rotation and rib cage asymmetry [1,2]. Etiologically, scoliosis is classified as congenital, neuromuscular, and idiopathic, with idiopathic scoliosis comprising approximately 85% of cases [3]. Idiopathic scoliosis is further subdivided based on age of onset into infantile (0–3 years), juvenile (4–9 years), and adolescent (≥10 years), with adolescent idiopathic scoliosis being the most prevalent subtype in Western literature [3,4].

Early detection of scoliosis is vital for minimizing complications and optimizing outcomes. Timely identification enables non-invasive interventions, such as bracing, to halt curve progression during growth, reducing surgical need [5]. Prompt management improves physical comfort, posture, and quality of life, reinforcing the importance of effective screening and early therapeutic support for long-term success [6]. School-based scoliosis screening programs potentially offer an effective strategy for early detection and timely intervention, particularly during critical growth phases [7]. These initiatives not only facilitate early management to prevent curve progression but also enhance spinal health awareness among students, families, and educators, promoting proactive care [8].

School-based scoliosis screening programs have been widely adopted across countries as an effective strategy for early detection and timely intervention [9]. For example, in the U.S., several states mandate school screenings during key growth phases [9]. Nations such as Singapore, Turkey, and Malaysia emphasize the cost-effectiveness and clinical value of these programs, while Greece highlights both benefits and implementation challenges [10]. Despite varying approaches, such initiatives aim to allow early non-invasive interventions and improve health outcomes in school-aged populations [11]. School-based scoliosis screening combines physical examination and imaging, with the Adam’s Forward Bend Test and scoliometer-based ATR measurement serving as key non-invasive tools [12]. Radiographic confirmation via spinal X-ray remains essential, with a Cobb angle ≥10° serving as the diagnostic benchmark for scoliosis [13].

Scoliosis is believed to affect 2–3% of adolescents globally, with adolescent idiopathic scoliosis (AIS) being the most common subtype, typically diagnosed between ages 10–15 and disproportionately affecting females with higher progression risk [14]. Congenital scoliosis (CS), caused by vertebral malformations, occurs in 0.5–1 per 1,000 live births, with regional variation; while females show higher overall prevalence, segmentation defects are more frequent in males [14]. Neuromuscular scoliosis (NMS) varies widely, affecting 20–75% of children with cerebral palsy and over 80% of those with Duchenne muscular dystrophy [15,16]. Syndromic scoliosis, linked to genetic disorders such as Marfan syndrome, NF1, Prader-Willi, and Rett syndrome, shows high prevalence (30–80%) with early onset and rapid progression, often worsening with age and motor decline [17].

Scoliosis remains a significant public health concern in Europe, affecting 1–3% of children and adolescents, with adolescent idiopathic scoliosis (AIS) being the most prevalent form [18]. Females are approximately 1.45 times more likely than males to develop scoliosis and face a greater risk of curve progression [19]. In Asia, scoliosis is an emerging public health concern, with adolescent idiopathic scoliosis (AIS) affecting 0.5% to 3% of youths, depending on regional and demographic factors [14]. In China, prevalence among children aged 5–18 is estimated at 0.79%, representing a substantial caseload [14]. The condition is more prevalent in females, with rates of 1.6% compared to 1.0% in males [20].

Scoliosis prevalence in Africa is poorly documented, though estimates suggest 2–3% of adolescents are affected, consistent with global trends [21]. In Ethiopia, radiograph-based studies reported a prevalence of 2.2% [22], while school-based screenings identified idiopathic scoliosis in 1.8% of children [23]. However, these studies were limited by their narrow scope, non-specialized imaging, and lack of standardized protocols. This study addresses gaps through a large-scale, standardized screening to better characterize the burden and distribution of scoliosis among Ethiopian schoolchildren.

Scoliosis screening in Ethiopia has not been well studied; we conducted this school-based screening project to assess the prevalence of scoliosis in schoolchildren and characterize the type of scoliosis they had. This provided us with insights into the epidemiology as well as the feasibility of conducting school screenings for this condition in this LMIC context.

## 2. Materials and methods

### 2.1. Study design and setting

A cross-sectional, school-based screening study was conducted from March 1, 2024, to June 15, 2025, in selected public primary schools across six Ethiopian areas: Addis Ababa, Hawassa, Wolaita, Weliso, Adama, and Jimma. Study sites were purposively selected based on their population size, accessibility, and logistical feasibility, especially considering the lack of previous scoliosis prevalence data in Ethiopia. The selected geographic areas limit the claim of national representativeness. The study encompassed 42 public elementary schools, with seven schools selected from each of the six areas. Using a multistage stratified cluster sampling technique, a minimum of 762 students aged 6–15 years were recruited from each school to ensure representative coverage across age groups and geographic locations. This study design facilitated broad geographic representation, enhancing the generalizability of findings across diverse Ethiopian settings. The selected areas varied in altitude, from 1,708 meters in Hawassa [24] to 2,355 meters in Addis Ababa [25], and in location distance from the capital city, ranging from 99 km (Adama) [26] to 349 kilometers (Jimma) [27].

### 2.2. Study source and population

The source population included all students enrolled in 42 public primary schools within the selected areas. The target population consisted of children aged 6–15 years who were attending these schools during the screening period. All students aged 6–15 years enrolled in the selected 42 public primary schools were included in the study. Exclusion Criteria included students who were unable to cooperate with Adam’s forward-bending test due to physical or cognitive limitations, as well as students or caregivers who declined to participate in the scoliosis screening.

### 2.3. Sampling procedure

A multistage stratified cluster sampling approach was employed to recruit participants. In the first stage, six Ethiopian areas: Addis Ababa, Adama, Hawassa, Wolaita Sodo, Jimma, and Weliso, were purposively selected based on accessibility, population size, and logistical feasibility. In the second stage, seven elementary schools were randomly chosen from each area, yielding a total of 42 schools. The third stage involved random selection of entire classrooms within each school, from which all students aged 6–15 years were invited to participate. A minimum of 762 students were enrolled per school to ensure age and grade-level diversity.

### 2.4. Screening procedure

Scoliosis screening was carried out among students aged 6–15 years across 42 public elementary schools in six Ethiopian areas: Addis Ababa, Adama, Hawassa, Wolaita Sodo, Jimma, and Weliso. The screening process was implemented in close collaboration with school administrators, teachers, and healthcare professionals to ensure logistical coordination and ethical compliance. Trained nurse professionals conducted the assessments using standardized protocols, including the Adam’s Forward Bend Test (AFBT) and scoliometer measurements. Students with ATR ≥ 7° were identified as suspected cases of scoliosis [11]. All data was uploaded to a secure, anonymized online database. All participants’ parents or caregivers were informed and provided written consent before screening. Students presenting with an angle of trunk rotation (ATR) ≥7° or a positive AFBT were referred to CURE Children’s Hospital of Ethiopia for further clinical evaluation and diagnostic confirmation.

The study employed Bunnell’s standardized Angle of Trunk Rotation (ATR) threshold of ≥7° as a referral criterion for further investigation. [11,23,28]. All children who screened positive received an information leaflet for their family to indicate the reason for referral for further investigation.

### 2.5. Radiographic procedures

Scoliosis severity was assessed using standardized standing postero-anterior spinal radiographs. Patients were positioned upright with arms at their sides and feet shoulder-width apart. Imaging was performed with calibrated digital X-ray equipment suitable for pediatric use, with exposure parameters minimized according to ALARA (As Low As Reasonably Achievable) principles [29]. Quality assurance included routine equipment checks and image verification. Each patient underwent a single exposure unless repeat imaging was required.

### 2.6. Cobb angle measurement protocol

Cobb angles were measured independently by two trained raters using standardized digital tools. Raters were blinded to participant information, and reliability was assessed through repeated measurements on a subset of radiographs. Both inter- and intra-rater agreement were evaluated, with intraclass correlation coefficients confirming acceptable reproducibility.

### 2.7. Statistical analysis

Data were managed and analyzed in Python 3.12 using Jupyter Notebook. The dataset was checked for completeness and accuracy, with outliers verified against original records. Descriptive statistics summarized demographic and clinical characteristics, with appropriate measures for continuous and categorical variables. Scoliosis prevalence was estimated both from field screening and radiographic confirmation, with 95% confidence intervals calculated using the Wilson method. Group differences were tested with chi-square/Fisher’s exact tests for categorical data and t-tests/Mann–Whitney U tests for continuous data. Logistic regression identified independent risk factors, adjusting for age and sex, and model fit was assessed with the Hosmer–Lemeshow test and VIF.

Correlations between trunk rotation and Cobb angle were examined using Pearson’s coefficient. Analyses employed standard Python libraries, with results presented in tables and figures.

#### 2.7.1. Cluster design effects and sampling weights.

A multistage stratified cluster sampling technique was used to recruit students; however, cluster design effects and sampling weights were not applied in the statistical analyses. All analyses were conducted on unweighted data, which may underestimate variance due to clustering. Consequently, prevalence estimates should be interpreted as representative of the sampled regions rather than nationally generalizable.

#### 2.7.2. Sensitivity analyses.

Sensitivity analyses were performed to evaluate the impact of attrition during referral by recalculating prevalence estimates and curve-type distributions under alternative assumptions about missing cases.

### 2.8. Data quality control

To ensure the reliability and accuracy of the data, all data collectors underwent standardized training in scoliosis assessment, study protocols, and ethical procedures. A pilot study was conducted to refine the tools and address any inconsistencies. Validated instruments were utilized to minimize measurement errors, and data collection was closely supervised. A double-entry system allowed for cross-verification of records, and a random subset of the data was reviewed for consistency. Ethical standards were rigorously maintained throughout the study.

### 2.9. Ethical considerations

The study protocol was reviewed and approved by the Institutional Review Board of Addis Ababa University, College of Health Sciences (Protocol Number: 010/24/Anat, Date: February 21, 2024). Before data collection, informed written consent was obtained from parents and verbal consent from school administrators and students. Privacy and confidentiality were maintained, and no personal identifiers were used in the analysis.

## 3. Result

The study screened a total of 32,000 children, of whom 48 were initially suspected to have scoliosis, resulting in an overall screening prevalence of 0.15% (95% CI: 0.11–0.20%)**.** Following referral and hospital confirmation, only 21 children were verified as true scoliosis cases, yielding a confirmed prevalence of 0.066% (95% CI: 0.04–0.10%)**.** This discrepancy between suspected and confirmed cases demonstrates that community-level screening may overestimate the prevalence of scoliosis, emphasizing the importance of diagnostic confirmation. The study found that approximately 7 per 10,000 children screened were confirmed to have scoliosis.

Among the 32,000 children screened, congenital scoliosis was the most common type, affecting 0.041% of the population, followed by idiopathic scoliosis (0.016% of the adolescent population age 10–15). Neuromuscular and syndromic scoliosis were rare, with prevalences of 0.006% and 0.003%, respectively. Overall, the findings indicate that congenital scoliosis contributes the largest share of scoliosis cases detected in this study, while other types occur at very low frequencies in the screened population.

As shown in Table 1, the screened children had a mean age of 11.8 years (range 6–15), with average height and arm span around 138 cm, reflecting proportional growth. Nutritional status varied, with a mean MUAC of 18.2 cm, and weights ranged from 15–55 kg. The average trunk rotation angle in these positively screening tests was 12.2°, exceeding the 7° threshold in most cases, indicating clinically relevant scoliosis and warranting further assessment.

**Table 1:** Descriptive statistics of anthropometric and screening characteristics among schoolchildren aged 6–15 years in Ethiopia with ATR ≥ 7°.

Predictor	Count	Mean	Std	Min	25%	Median	75%	Max
Child Age	48	11.79	2.73	6	10.75	12	14	15
Child’s Height	48	138.12	18.04	100	128	143	150	175
Child’s arm span from fingertip to fingertip	48	138.77	19.76	100	125	144	151.25	185
MUAC	48	18.19	3.13	13	15.88	18	20	25
Child’s Weight	48	32.71	11.38	15	24	32.5	40	55
ATR (Angle of Trunk Rotation)	48	12.21	5.26	7	9	10	14	32

As indicated in Table 2, among the 48 children screened, congenital scoliosis was the most common type (27.1%), followed by idiopathic (10.4%), with smaller proportions of neuromuscular (4.2%) and syndromic cases (2.1%). A notable share either had no scoliosis (14.6%), refused further assessment (22.9%), or were uncontactable (10.4%), limiting full diagnostic confirmation. Slightly more than half of the participants were male (54.2%), and most were in middle primary grades (43.8%). Parental occupation was dominated by self-employment (50%), with unemployment accounting for 29.2%. Clinically, scoliosis was most often thoracic (52.1%), followed by lumbar (25%) and thoracolumbar (22.9%), with rib humps more frequent on the right side (66.7%).

**Table 2:** Distribution of scoliosis types, demographic characteristics, and screening features among schoolchildren (n = 48).

Variable	Category	Count	Percentage (%)
**Type of scoliosis**	Congenital scoliosis	13	27
	Idiopathic scoliosis	5	10
	Neuromuscular scoliosis	2	4
	Syndromic scoliosis	1	2
	Not scoliosis (normal/other) (normal child, Cobb<10, CP not scoliosis, CP + LLD, LLD no scoliosis)	7	14
	Refused	11	22
	Uncontactable	5	10
**Sex**	Male	26	54
	Female	22	45
**Grade at school**	1-3 Grade	16	33
	4-6	21	43
	7-8	8	16
	Others	3	6
**Occupation of a working parent**	Self-employed	24	50
	Unemployed	14	29
	Others	10	20
**Site of the scoliosis**	Thoracic	25	52
	Lumbar	12	25
	Both (thoraco-lumbar)	11	22
**Side of the rib hump**	Right	32	66
	Left	16	33

In Table 3, we present data on 21 confirmed cases of scoliosis. The average age of the children is 11.2 years, with a mean height of 131.7 cm and an arm span of 135.2 cm, both of which indicate generally proportional growth. Their nutritional status varies, showing a mean Mid-Upper Arm Circumference (MUAC) of 17.3 cm and an average weight of 28.1 kg. Clinically, all children displayed a significant trunk rotation, with a mean Angle of Trunk Rotation (ATR) of 16°. The Cobb angles exhibited considerable variation, ranging from 11° to 97°, with a mean of 47.4°, indicating the presence of deformities ranging from mild to severe. Additionally, the average thoracic height is measured at 18.8 cm, the median height at 22.1 cm, and the percentage of thoracic loss is 15.2%, highlighting the measurable structural impact of scoliosis on growth and thoracic development.

**Table 3:** Descriptive statistics of anthropometric and clinical parameters among schoolchildren diagnosed with scoliosis.

Variable	Mean (SD)	Min	Max	Median	25th – 75th Percentile
**Age (years)**	11.19 (2.29)	7	15	12	9–13
**Height (cm)**	131.71 (15.05)	103	155	136	121–143
**Arm span (cm)**	135.24 (15.98)	102	160	136	122–150
**MUAC (cm)**	17.26 (2.90)	13	25	18	15–19
**Weight (kg)**	28.05 (9.70)	15	50	26	20–35
**ATR (°)**	15.95 (5.74)	10	32	15	11–19
**Cobb angle (°)**	47.38 (28.93)	11	97	37	24–68
**Thoracic height (cm)**	18.79 (2.74)	13.4	23	18.9	18–21
**Median height (cm)**	22.13 (1.98)	18.8	24.8	22.8	20–24.3
**% Thoracic loss**	15.21 (9.78)	1.6	32.02	15.79	5.7–21.81

As shown in Table 4, among the 21 confirmed scoliosis cases, males predominated (66.7%), and most were in grades 1–6 (85.8%). Congenital scoliosis was the leading type (61.9%), followed by idiopathic (23.8%), neuromuscular (9.5%), and syndromic (4.8%). The thoracic spine was most frequently affected (52.4%), with lumbar and thoraco-lumbar involvement each accounting for 23.8%. Rib humps were more common on the right side (61.9%), and severity ranged from mild (28.6%) to very severe (19.0%), reflecting a broad clinical spectrum. Overall, congenital scoliosis in male school-aged children emerged as the predominant pattern, underscoring the need for early detection and tailored management.

**Table 4:** Summary of demographic and anatomical features in schoolchildren diagnosed with scoliosis.

Variable	Category	Count	Percentage (%)
**Sex**	Male	14	66.7
	Female	7	33
**Grade at school**	1–3	9	42.9
	4–6	9	42.9
	7–8	1	4.8
	Others	2	9.5
**Type of scoliosis**	Congenital	13	61.9
	Idiopathic	5	23.8
	Neuromuscular	2	9.5
	Syndromic	1	4.8
**Site of scoliosis**	Thoracic	11	52.4
	Lumbar	5	23.8
	Thoraco-lumbar	5	23.8
**Side curve**	Right	13	61.9
	Left	8	38.1
**Severity**	Mild (Cobb = 10–24)	6	28.6
	Moderate (Cobb = 25–40)	5	23.8
	Severe (Cobb = 41–80)	6	28.6
	Very severe (Cobb >80)	4	19

**Fig 1:**
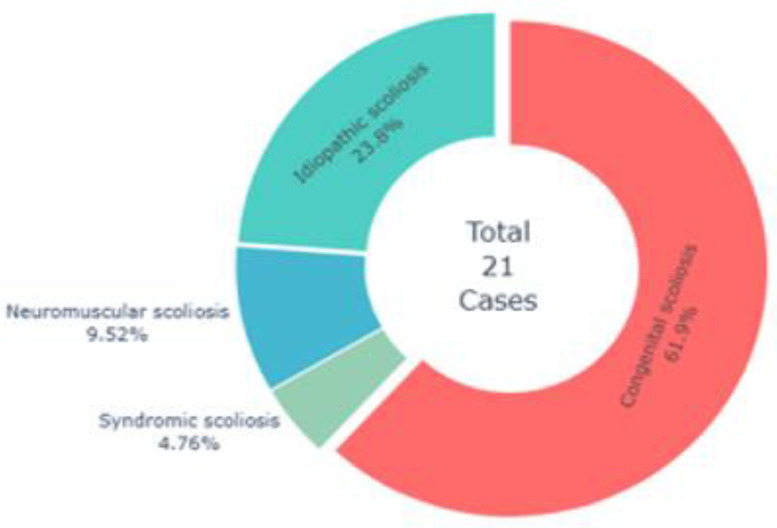
Distribution of scoliosis cases by curve type.

Congenital scoliosis was the most common type (61.9%), followed by idiopathic (23.8%), with fewer neuromuscular (9.5%) and syndromic cases (4.8%). Overall, the distribution highlights congenital malformations as the leading cause of scoliosis in this study (Fig 1).

**Fig 2:**
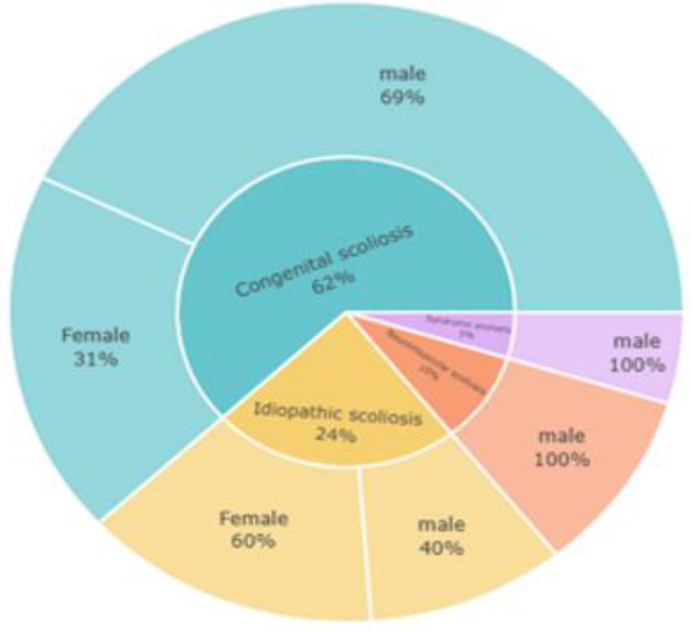
Distribution of scoliosis types by sex.

As shown in Fig 2, the sunburst chart depicts scoliosis types by sex. Congenital scoliosis was most common (62%), with a clear male predominance (69%), while idiopathic scoliosis accounted for 24% and was more frequent among females (60%). Neuromuscular (10%) and syndromic scoliosis (5%) were less frequent and observed only in males. Overall, congenital scoliosis predominated in males, whereas idiopathic scoliosis was more frequent in females.

**Fig 3:**
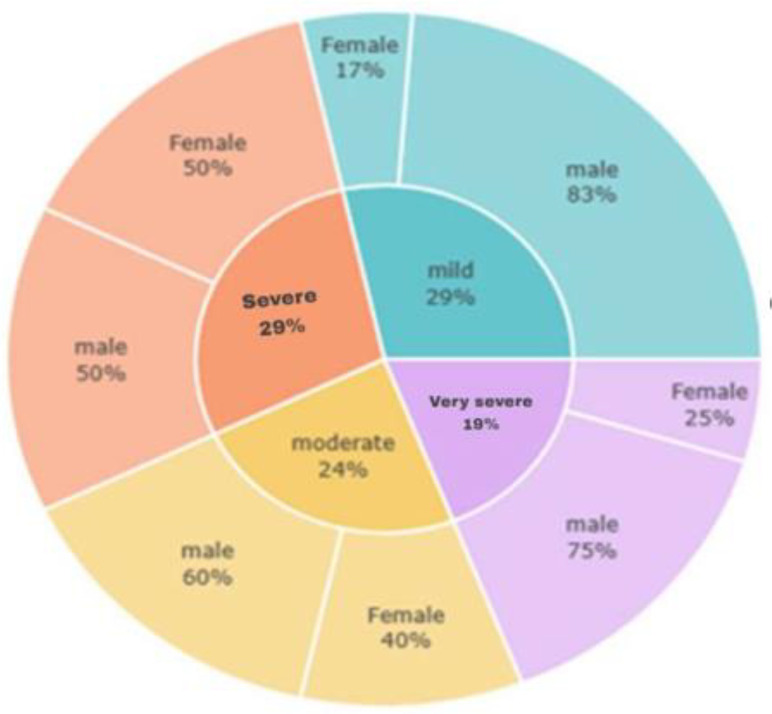
Distribution of scoliosis severity by sex (n = 21).

As shown in Fig 3, scoliosis severity varied across the 21 confirmed cases. Mild scoliosis accounted for 29% of cases, with a marked male predominance (83%). Severe scoliosis also comprised 29% but was evenly distributed between sexes (50% each). Moderate scoliosis represented 24%, occurring more often in males (60%), while very severe scoliosis was the least common (19%) and predominantly male (75%). Overall, males were more frequently affected across most severity levels, particularly in mild and very severe categories, whereas females were more evenly represented in severe cases.

**Fig 4:**
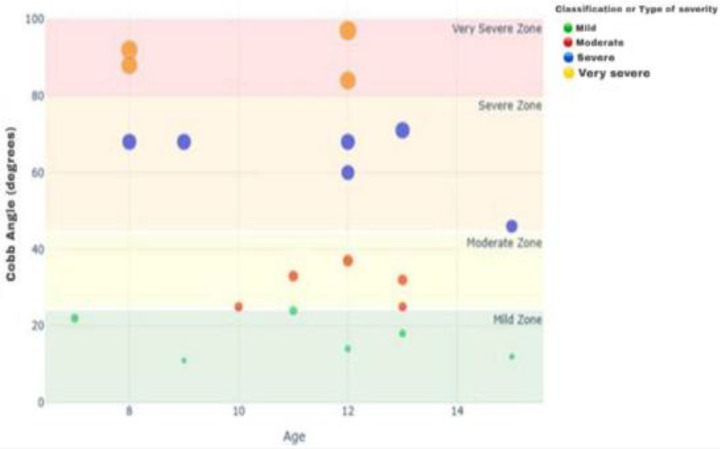
Scoliosis severity by Cobb angle across age groups.

As shown in Fig 4, Cobb angle measurements demonstrated a wide spectrum of scoliosis severity across ages. Mild cases (≤24°) were distributed across all age groups, while moderate cases (25°–44°) clustered mainly between 10–13 years. Severe scoliosis (45°–80°) occurred across a broad age range, and very severe cases (>80°) were concentrated in younger children (ages 8 and 12). These findings highlight that scoliosis severity varies with age, with advanced deformities evident even in early school years, underscoring the need for early detection and monitoring.

**Fig 5:**
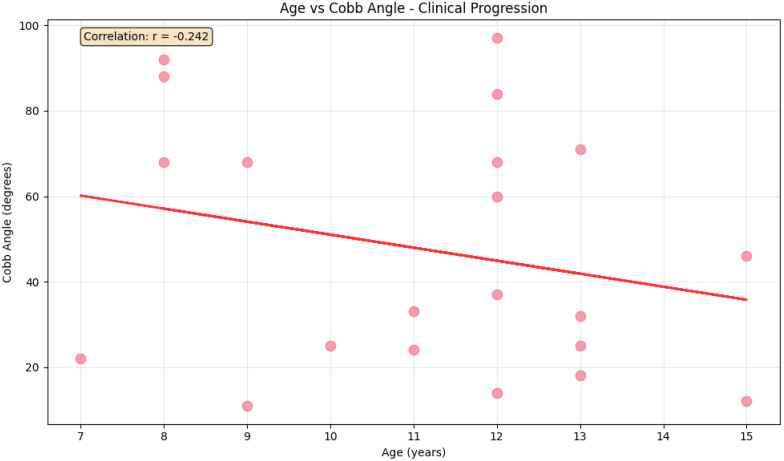
Age-related variation in Cobb angle among children with scoliosis.

As shown in Fig 5, the scatter plot demonstrates a weak negative correlation (r = –0.242) between age and Cobb angle, indicating a slight decrease in curvature severity with increasing age. This trend may reflect slower progression or clinical intervention, but the modest correlation suggests that additional factors such as growth, genetics, and treatment also influence scoliosis development.

**Fig 6:**
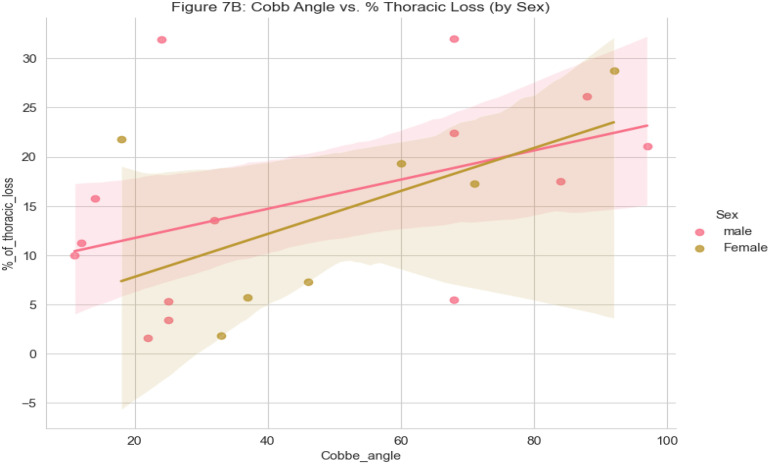
Relationship between Cobb angle and thoracic loss by sex.

The Cobb angle was positively associated with thoracic height loss in both sexes, indicating that greater spinal curvature corresponded to increased thoracic loss. The relationship was stronger in males, reflected by the steeper regression slope, suggesting a greater impact of curvature on thoracic anatomy compared to females. Narrow confidence intervals around the regression lines support the consistency of these findings, highlighting potential sex-based differences relevant for clinical assessment and management (Fig 6).

**Fig 7:**
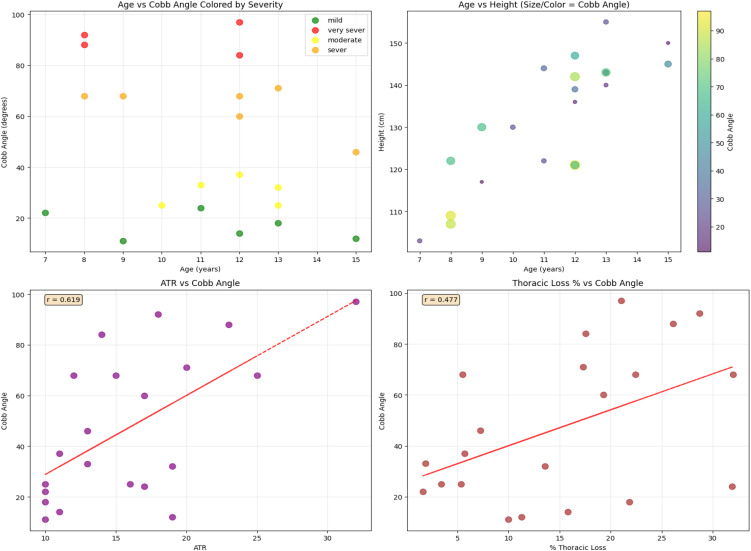
Relationships between Cobb angle and key clinical parameters in children’s scoliosis.

The Cobb angle demonstrated distinct relationships with key clinical factors. It tended to increase with age and severity, while appearing across all age and height ranges, indicating curvature is not directly dependent on stature. A strong positive correlation was observed between Cobb angle and ATR (r = 0.613), confirming ATR as a reliable severity marker. Thoracic loss also showed a moderate positive correlation (r = 0.477), suggesting that greater curvature contributes to reduced thoracic volume. Collectively, these plots highlight the progression and clinical impact of scoliosis (Fig 7).

**Fig 8:**
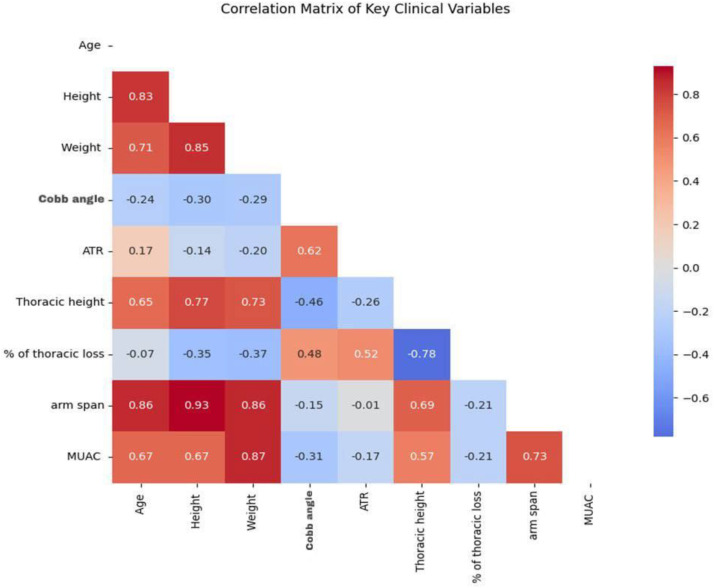
Correlation matrix of key clinical variables in children’s scoliosis.

As shown in Fig 8, the correlation matrix highlights key clinical relationships. Height strongly correlated with arm span (r = 0.93) and thoracic height (r = 0.81). Cobb angle was moderately associated with ATR (r = 0.61) and thoracic loss (r = 0.48), but negatively correlated with thoracic height (r = –0.46). Age also showed a moderate positive correlation with Cobb angle (r = 0.41). Thoracic height and thoracic loss were strongly inversely related (r = –0.78), underscoring the structural impact of scoliosis. Overall, these patterns emphasize how spinal curvature influences trunk rotation and thoracic anatomy.

## 4. Discussion

This study represents the first nationally representative effort to screen for the prevalence of scoliosis in schools across different sites of Ethiopia, leveraging a large sample size and a monitoring period extending over more than a year. It enabled both cross-sectional assessments and the analysis of temporal trends. Conducted in six geographically diverse areas, this research stands as the most comprehensive epidemiological investigation of scoliosis among Ethiopian primary schoolchildren aged 6–15 years, and found an initial scoliosis prevalence of 0.15% (95% CI: 0.11–0.20%). This finding is consistent with other study reports of screening-based prevalence rather than radiographically confirmed diagnoses using comparable screening protocols in Singapore (0.15%) [29], Norway [30] (0.13%), Turkey (0.13%) [31].

The point prevalence of suspected scoliosis in the Ethiopian school-based screening program was higher than reported in Greece (0.11%) [32]. This elevated prevalence likely reflects the use of standardized physical examination protocols, the inclusion of a broad age range (6–15 years), and regional differences in skeletal maturity [33], nutritional status and pubertal timing [34]. These findings underscore the importance of locally adapted screening strategies tailored to demographic and developmental contexts.

The prevalence of suspected scoliosis in the present study is lower than that reported in China (2.37%) [35], Brazil (1.5%) [36], Korea (0.54%) [37], Turkey (1.8%) [38], US (4.1%) [39], and a prior small school screening study Ethiopia 1.8% [23]. Multiple contextual factors may account for the lower scoliosis prevalence observed. Methodologically, the use of a conservative referral threshold (ATR ≥ 7°) may have limited case detection compared to broader criteria used elsewhere. Additionally, active lifestyles and minimal exposure to postural stressors likely contribute to the observed disparity, underscoring this study’s value in addressing a critical data gap in Sub-Saharan Africa [40]. Based on clinical and radiological confirmation, the prevalence of scoliosis among Ethiopian schoolchildren was established at 0.066% (95% CI: 0.04–0.10%), providing a validated national estimate. This present finding is consistent with studies in Japan (0.077%) [41], the US (0.05%) [39], and Singapore (0.05%) [42]. However, the confirmed scoliosis prevalence in Ethiopia (0.066%) is a higher rate than in Singapore (0.02%) [29], Japan (0.04%) [43], and Greece (0.04%) [44], which may likely be due to methodological and demographic differences. Broader age inclusion and standardized radiographic confirmation in the Ethiopian study enhanced early-stage detection, while lower rates elsewhere may reflect narrower screening criteria.

The confirmed scoliosis prevalence in the present Ethiopian study (0.066%) is notably lower than the global estimates, which range from 0.47% to 5.2% in population-based studies of many countries, such as Germany [20], Korea [45], Greece [46], England [47], Singapore [48], Turkey [49], Saudi Arabia [50], China [51], USA [52]. It also falls below previously reported rates from other African nations, including earlier Ethiopian studies (1.8%–2.2%) [49], (1.8%) [22], Ghana (3.3%) [53], South Africa (8.3%) [54]. The comparatively low scoliosis prevalence reported in this Ethiopian study likely stems from a combination of methodological consistency, such as the application of strict diagnostic criteria and selective radiographic confirmation, and systemic barriers to early detection, including referral attrition, sociocultural beliefs, and limited geographic reach. Variations from international findings may also reflect differences in age profiles, screening sensitivity, and healthcare accessibility, rather than representing a genuine epidemiological divergence.

The mean age of scoliosis diagnosis in this study was 11.2 years (SD ± 2.3), which is consistent with reports from China [35], and Ethiopia [23], but slightly younger than the averages noted in Norway [30], Turkey [49], Côte d’Ivoire [55]. This suggests that Ethiopia’s school-based screening program facilitates early detection, enabling timely conservative management. Anthropometric data, mean height of 131.7 cm, arm span of 135.2 cm, weight of 28.1 kg, and MUAC of 17.3 cm, indicate generally proportional growth with nutritional variability, potentially influencing musculoskeletal development. These patterns parallel findings from China [35].

The mean Cobb angle in this Ethiopian cohort (47.4° ± 28.9°) was markedly higher than values reported in China (12.9° ± 4.1° [56], Korea (below 20°) [45], Côte d’Ivoire (35.2° ± 10.71°) [55], Ethiopia (2.27 ± 6.320) [22], and Brazil (17.9° ± 28.9) [36]. likely reflecting delayed detection and limited access to early screening. This contrasts with the study of hospital-based data from Finland [57], which often reports preoperative angles exceeding 90°, highlighting differences in case referral patterns. The current Ethiopian findings underscore the importance of early detection in resource-limited settings where surgical options are limited.

The mean thoracic height in the Ethiopian study (18.8 cm) was lower than U.S. benchmarks achieved through early surgical intervention (25.2–26.1 cm) [58], reflecting delayed detection and limited access to growth-preserving care. Nutritional and environmental factors may further contribute to thoracic underdevelopment in early-onset cases in Japan. The observed thoracic loss (15.2% ± 9.8) exceeded U.S. benchmarks for growth-preserving interventions (5–12%) [58]. but was lower than the > 20% reported in Turkish thoracic insufficiency syndrome [59]. Overall, these findings highlight the substantial structural impact of scoliosis and reinforce the need for scalable screening and early intervention to reduce long-term cardiopulmonary risks.

In this Ethiopian study, scoliosis was confirmed with a male predominance of 66.7%, contrasting global trends of female dominance in adolescent idiopathic scoliosis. This pattern is consistent with age-specific findings from Turkey [18], where mild scoliosis is more common in younger males. Contributing factors may include early detection of congenital and neuromuscular cases, school attendance dynamics, and greater clinical visibility among boys. These results highlight the importance of age-sensitive screening and context-specific data to inform national referral strategies.

In this Ethiopian school-based screening, congenital scoliosis accounted for 61.9%, a rate markedly higher than global averages, where idiopathic scoliosis predominates. Although lower than Indian clinical reports (84.6–92.7%) [60], it exceeded figures from Israel (21.1%) [61] and Côte d’Ivoire (15.1%) [55]. This elevated prevalence likely reflects improved detection through targeted school screening in underserved areas, alongside limited access to early imaging that delays diagnosis until deformities are clinically evident. Possible etiological contributors include maternal folate deficiency and teratogenic exposures. By emphasizing vertebral malformations, diagnostic sensitivity was enhanced [62]. These findings highlight the importance of early detection and etiological research in low-resource settings.

The 23.8% prevalence of idiopathic scoliosis in this study is markedly higher than regional and global estimates, such as 8.2% in South Africa [54], and 1.8% in Ethiopia. [23], but remains lower than the 74.5% reported in Côte d’Ivoire [55]. Variability across international studies in China [63], Turkey [64], US [65], likely reflects differences in methodology, diagnostic thresholds, and population characteristics. The higher rate in this study may stem from broader inclusion criteria and population-specific factors. Neuromuscular (9.5%) and syndromic (4.8%) scoliosis rates align with global data in Turkey [18], China [66], and the Netherlands [67], reinforcing the need for subtype stratification and screening protocols that account for systemic contributors.

Thoracic curves were the most common in this study (52.4%), with a predominance of right-sided alignment (61.9%), consistent with findings from Côte d’Ivoire [55], Japan [68], and Canada [69]. The current study revealed a higher or lower rate than the previous studies in China [14], Japan [43], Korea [45], Turkey [38], and Singapore [42]. The 66.7% rate of right convexity aligns with global trends, including reports from Côte d’Ivoire [55], Chile [70], and Turkey [71]. This pattern may indicate that during adolescence, there is a tendency for age-related vertebral rotation to favor the right side.

The severity distribution in this finding, mild (29%), moderate (24%), severe (28%), and very severe (19%), reflects broader variation than typically reported in population-based studies. Males were disproportionately represented in mild (83%) and very severe (75%) categories, aligning with findings from Greece [72], but contrasting global trends where females predominate in moderate to severe idiopathic scoliosis reported in China [66], Korea [73], Norway [30]. These patterns may reflect regional screening practices, age profiles, and biological factors, underscoring the need to contextualize severity and sex distribution within local epidemiological frameworks.

The decline in Cobb angle with age observed in this study aligns with findings from South Africa [33], suggesting reduced progression near skeletal maturity. However, the weak correlation indicates that age alone is not predictive, with factors like curve type, genetics, and treatment history influencing progression. Males exhibited a steeper scoliosis-related thoracic height loss per Cobb angle unit, consistent with data from Turkey [59], Croatia [74], pointing to sex-linked anatomical and biomechanical differences. These findings highlight the need for sex-specific monitoring and individualized treatment approaches.

A moderate negative correlation between Cobb angle and thoracic height (r = –0.46), and a stronger association with percentage thoracic loss (r = –0.78), indicates that increasing curvature compresses vertical thoracic growth. Consistent with findings from Turkey [59], China [75]. These results underscore growth-phase vulnerability and support incorporating thoracic metrics into early-onset scoliosis assessments to guide timely intervention. Contrasting evidence from Poland [76] challenges reliance on Cobb angle alone to estimate thoracic height loss, emphasizing the influence of trunk morphology, curve flexibility, and rotational deformity. These findings highlight the complexity of scoliosis-related growth impairment and support multifactorial assessment protocols that incorporate thoracic metrics, curve rigidity, and age-specific growth potential.

This study offers pivotal epidemiological insights into scoliosis among Ethiopian school-aged children, notably revealing a high prevalence of congenital scoliosis and male predominance, findings that diverge from global patterns and suggest population-specific developmental and genetic influences. These unique characteristics highlight the need for early, context-sensitive screening strategies, particularly in resource-constrained settings. The observed age-specific progression, sex-based structural differences, and strong radiographic-functional correlations underscore the multifactorial nature of scoliosis and support the adoption of integrated assessment protocols. Policy efforts should prioritize school-based screening, diagnostic infrastructure, and referral systems for scoliosis. Clinically, the results support multifactorial assessment approaches that integrate radiographic, anthropometric, and developmental indicators to guide individualized care. Future research should investigate the genetic and biomechanical basis of scoliosis in this population and evaluate long-term outcomes of early intervention. By contributing rare regional data, this study helps bridge a critical gap in global scoliosis literature and informs context-specific public health and clinical strategies for East African children.

Although attrition during referral may have introduced potential bias, sensitivity analyses demonstrated that prevalence estimates and curve-type distributions remained stable, supporting the robustness of our findings. The most notable finding in this research is that there are relatively few adolescent idiopathic cases detected in the school-screened Ethiopian population. Adolescent idiopathic scoliosis is the most prevalent group in the majority of the studies published. There is no obvious reason for this discrepancy, and this would be a topic for further investigation. However, the low prevalence of idiopathic adolescent scoliosis has implications. A national school screening programmed to identify and treat early idiopathic cases with a brace would not yield a significant number of treatable idiopathic.

## 5. Conclusion

This study provides the first comprehensive epidemiological profile of scoliosis among Ethiopian schoolchildren aged 6–15 years, revealing a low overall prevalence but a clinically significant burden driven by congenital scoliosis, early onset, and male predominance in severe cases. The predominance of thoracic involvement and strong correlations between Cobb angle, thoracic height loss, and trunk rotation highlight the structural impact of scoliosis and the need for individualized, sex-specific monitoring. By establishing a reliable baseline for national surveillance, these findings help fill a critical gap in local data and underscore the importance of early detection and sustained monitoring for severe cases. Given the rarity of scoliosis and the limited applicability of non-invasive treatment, routine school-based screening programs are not warranted in Ethiopia; instead, resources should be focused on strengthening diagnostic and referral pathways for clinically evident cases to ensure timely access to specialized care.

## Abbreviations

AFBT: Adam`s forward bend test
AIS: Adolescent idiopathic scoliosis
ALARA: As Low As Reasonably Achievable
ATR: Angle of trunk rotation
BMI: Body mass index, CS, Congenital scoliosis
CI: Confidence interval
LMIC: Low and middle-income. countries, NMS, Neuromuscular scoliosis
SD: Standard deviation VIF, Variance inflation factor
